# The development of a faecal incontinence core outcome set: an international Delphi study protocol

**DOI:** 10.1007/s00384-021-03865-2

**Published:** 2021-02-02

**Authors:** Sadé Assmann, Daniel Keszthelyi, Jos Kleijnen, Merel Kimman, Foteini Anastasiou, Elissa Bradshaw, Emma Carrington, Giuseppe Chiarioni, Yasuko Maeda, Jean Muris, Daniel Pohl, Mona Rydningen, Carolynne Vaizey, Stephanie Breukink

**Affiliations:** 1 Department of Surgery and Colorectal Surgery, Maastricht University Medical Centre, Maastricht, The Netherlands; 2 Division of Gastroenterology-Hepatology, Department of Internal Medicine, Maastricht University Medical Centre, Maastricht, The Netherlands; 3 School of Nutrition and Translational Research in Metabolism (NUTRIM), Maastricht University, Maastricht, The Netherlands; 4 School for Oncology and Developmental Biology (GROW), Maastricht University, Maastricht, The Netherlands; 5 Department of Clinical Epidemiology and Medical Technology Assessment, Maastricht University Medical Centre, Maastricht, The Netherlands; 6 4rth TOMY – Academic Primary Care Unit Clinic of Social and Family Medicine, University of Crete, Heraklion, Greece; 7 Community Gastroenterology Specialist Nurse, Royal Free Hospital, London, England; 8 Surgical Professorial Unit, Department of Colorectal Surgery, St Vincent’s University Hospital, Dublin, Ireland; 9 Division of Gastroenterology of the University of Verona, AOUI Verona, Verona, Italy; 10 Center for Functional GI and Motility Disorders, University of North Carolina at Chapel Hill, Chapel Hill, NC USA; 11 Department of Surgery and Colorectal Surgery, Western General Hospital, Edinburgh, UK; 12 Department of General Practice, Care and Public Health Research Institute, Maastricht University, Maastricht, The Netherlands; 13 Department of Gastroenterology and Hepatology, University Hospital of Zurich, Zurich, Switzerland; 14 Department of Gastrointestinal Surgery, University Hospital of North Norway, Tromsø, Norway; 15 Norwegian National Advisory Unit on Incontinence and Pelvic Floor Health, Tromsø, Norway; 16 St Mark’s Hospital, The National Bowel Hospital, London, UK

**Keywords:** Faecal incontinence, Core Outcome Set, Protocol, Patient interviews, Systematic review

## Abstract

**Purpose:**

Faecal incontinence (FI) is estimated to affect around 7.7% of people. There is a lack of uniformity in outcome definitions, measurement and reporting in FI studies. Until now, there is no general consensus on which outcomes should be assessed and reported in FI research. This complicates comparison between studies and evidence synthesis, potentially leading to recommendations not evidence-based enough to guide physicians in selecting an FI therapy. A solution for this lack of uniformity in reporting of outcomes is the development of a Core Outcome Set (COS) for FI. This paper describes the protocol for the development of a European COS for FI.

**Methods:**

Patient interviews and a systematic review of the literature will be performed to identify patient-, physician- and researcher-oriented outcomes. The outcomes will be categorised using the COMET taxonomy and put forward to a group of patients, physicians (i.e. colorectal surgeons, gastroenterologists and general practitioners) and researchers in a Delphi consensus exercise. This exercise will consist of up to three web-based rounds in which participants will prioritise and condense the list of outcomes, which is expected to result in consensus. A consensus meeting with participants from all stakeholder groups will take place to reach a final agreement on the COS.

**Discussion:**

This study protocol describes the development of a European COS to improve reliability and consistency of outcome reporting in FI studies, thereby improving evidence synthesis and patient care.

**Trial registration:**

This project has been registered in the COMET database on the 1st of April 2020, available at http://www.comet-initiative.org/Studies/Details/1554. The systematic review has been registered on the PROSPERO database on the 31st of August 2020, available at https://www.crd.york.ac.uk/PROSPERO/display_record.php?RecordID=202020&VersionID=1381336.

## Introduction

Faecal incontinence (FI) is a common anorectal problem which is defined by the ROME IV criteria as having recurrent uncontrolled passage of faecal matter for a minimum of 3 months [1]. The prevalence of FI is estimated to be around 7.7% [2]. The most important risk factors for developing FI include advanced age, previous rectoanal or obstetric surgery, obstetric trauma and/or neurological disorders [1]. These risk factors may result in a failure in the interaction between stool consistency, function of the rectal reservoir, stability of the pelvic floor, function of the anal sphincter complex and neurological function, resulting in FI [3]. Faecal incontinence can cause the development of secondary medical morbidities such as skin deterioration and has a negative impact on a person’s quality of life (QoL) [4, 5]. FI can result in embarrassment, low self-esteem, social isolation, avoidance of activities and depression and can have a negative impact on intimate relationships [5–7].

Numerous different treatment options are available for FI, but at present there is no uniform approach to which treatment a patient should receive [8]. Currently, there is no general consensus on which outcomes should be assessed in FI interventional studies [8, 9]. Several systematic reviews reporting on FI highlighted the lack of uniform outcome definition, measurement and reporting [10–14]. These deficiencies limit the ability to compare studies, subsequently complicating evidence synthesis, which in turn hinders the formulation of high-level evidence recommendations in future guidelines [14–16]. This problem can be alleviated by standardising the reporting of outcomes in clinical studies.

The development of a Core Outcome Set (COS) for FI will reduce heterogeneity in outcome reporting through creating a standardised minimum set of outcomes which researchers are encouraged to report on when performing FI intervention studies [17]. Improving homogeneity of reported outcomes, and thus improving comparison between studies, is expected to lead to more evidence-based and uniform FI treatment recommendations [17]. Furthermore, outcomes which are reported on in research are not always those which the patients regard as important or relevant outcomes [18]. Including patients with FI in this process will result in outcomes more relevant to the patient. This paper describes the protocol for the development of a COS which can be used in interventional studies for FI, evaluating treatment effectiveness.

## Methods

This study uses a stepwise approach to develop a COS as recommended by the guidelines for COS development, published by the Core Outcome Measures in Effective Trials (COMET) initiative [17]. The first step in creating the COS will be the identification of the most important outcomes for patients, researchers and physicians. These outcomes will be identified through patient interviews and a systematic review of the literature [19]. In the second step, patients, researchers and physicians are able to prioritise these outcomes and eventually converge towards a consensus opinion on the importance of these outcomes through the use of online-based Delphi surveys. Up to three Delphi rounds will take place, unless consensus has been reached after two rounds. The third step is a consensus meeting with representatives from each stakeholder group to decide on a definitive COS.

### Step 1: Identification of outcomes

Patient interviews will be conducted and analysed to identify outcomes which are relevant to patients with FI. Furthermore, a systematic review will be performed to identify reported outcomes frequently assessed in FI intervention studies. This systematic review will be reported according to the Preferred Reporting Items for Systematic Review and Meta-analyses (PRISMA) statement [20].

#### Patient interviews

Semi-structured interviews will be conducted in a minimum of two countries to explore outcomes important to patients. Interviews will be conducted with patients suffering from FI. Participant recruitment and interviews will be conducted until data saturation has been reached (i.e. at the point that no more new outcomes are mentioned by participants, two more interviews will be held to ensure data saturation had truly been reached). Semi-structured interviews will allow the patients to go into as much or as little detail as desired; hence, emphasis will be put on what is most important to the patient. Interviews are expected to take 30 to 45 min and will be audio recorded and transcribed verbatim. Two authors will independently analyse all of the interviews for content and list the outcomes which the patients have identified as important.

##### Participant recruitment

Participants will be recruited through gastroenterology and general surgery outpatient clinics and/or through a pelvic floor physiotherapist. Any patients with FI aged between 18 and 75 are eligible for participation. The estimated sample size for the interviews is 20 patients but interviews will continue until data saturation has been reached. Patients will be informed about the study through the physician or physiotherapist treating them and, if interested in participating, will receive further information via post or e-mail, depending on patient preferences.

Patients will be asked to contact the researcher to make an appointment for an interview if they are interested after reading the additional information. The patient will be asked for permission to be contacted by the researcher in case of no response 7 days after the initial physician/physiotherapist appointment. Participants will be asked to sign an informed consent form prior to the interview.

#### Systematic review

##### Search strategy

A literature search of MEDLINE (Ovid, supplemented by a recent PubMed search), Embase (Ovid) and the Cochrane Library (Cochrane Database of Systematic Reviews and Cochrane Controlled Register of Trials) will be conducted to identify relevant randomised controlled trials (RCTs), observational studies with control groups and systematic reviews examining treatment outcomes of first-line (e.g. diet and lifestyle changes, bulking agents, pelvic floor exercises), second-line (e.g. transanal irrigation) and/or surgical procedures (e.g. sacral neuromodulation, sphincter repair) in adult patients with FI. The search terms which will be used include ‘faecal incontinence’, any ‘treatment’ terms (e.g. therapy, surgery) and any ‘outcome’ terms (e.g. outcome, effectiveness) as well as any synonyms and spelling variations of these terms. The search will be limited to RCTs, observational studies with control groups and systematic reviews conducted in human adults, published from 2000 onward, with no language restrictions. The search strategy will be reviewed by an information specialist, prior to carrying out the full search.

##### Study selection

Any RCTs, observational studies with control groups and systematic reviews performed in humans can be included in the review. Furthermore, included systematic reviews will be scanned for relevant individual studies to be included in this review. Titles and abstracts of potentially relevant articles will be screened, followed by full text screening to assess study eligibility. Both abstract and title screening as well as full text screening will be performed independently by two reviewers using predetermined in- and exclusion criteria. Any disagreement on study eligibility will be resolved through discussion by the two reviewers with referral to a third senior author if necessary.

##### Data extraction

For each study, a predefined data extraction form will be filled out which includes author name, publication date, characteristics of the study, primary and secondary outcomes and outcome definitions, instruments used to measure outcomes and timing of assessment. Two reviewers will independently extract all data verbatim from the source manuscript and discuss results to ensure that the data forms are complete. No risk of bias assessment will be performed as the aim of the systematic review is only to explore which outcomes are reported on in FI intervention studies.

#### Categorisation of candidate outcomes

All identified outcomes established through the patient interviews and systematic review will be categorised into a maximum 38 outcome domains within five core areas as per the taxonomy published by the COMET initiative [21]. Prior to categorisation, any duplicate outcomes will be removed. Furthermore, it is likely that some outcomes will be the same but will have been defined or measured in various ways in different publications. An important step is to group these different definitions together under the same outcome name. The outcomes will then be formatted into questions to be used in the Delphi process. The questionnaire in the Delphi survey will be pilot tested by members of the steering group.

### Step 2: Delphi surveys

A Delphi process will be used to reach consensus on outcomes which should be included in the COS for FI. Up to three Delphi rounds will be conducted which can be filled in anonymously online by participants. The anonymity ensures that participants are not persuaded by dominant individuals when giving their responses and there is no group pressure for conformity [17, 22]. Furthermore, the online aspect allows for a large number of participants from multiple countries to participate in the process [17].

#### Participants

Patients with FI, healthcare professionals (i.e. colorectal surgeons, gastroenterologists and general practitioners) treating patients with FI and clinical researchers who have conducted research in FI will be invited to participate in the Delphi process.

The colorectal surgeons, gastroenterologists and general practitioners will be recruited internationally through the European Society of Coloproctology (ESCP), United European Gastroenterology (UEG) Society, the European Society of Neurogastroenterology and Motility (ESNM) and the European Society for Primary Care Gastroenterology (ESPCG) respectively. PubMed will be used to search for any clinical researchers who have conducted research in FI and who have not yet been recruited through any of the international societies. Patients will be identified through their medical records by their treating physician and will be recruited from a minimum of three different countries.

#### Delphi rounds

All outcomes which are found through the patient interviews and systematic review will be combined. Questions will be formulated to assess the perceived importance of each outcome.

Both the lay and medical terms will be used in the survey to ensure all stakeholders can understand each outcome. The wording patients use in the interviews can be used to label and explain outcome items in the survey to further ensure that the outcomes are understandable and accessible for patients [17]. Surveys will be translated for the patient groups for each of the included countries.

Participants will be asked to score each outcome on a 9-point Likert scale based on how important they consider the outcome to be in evaluating effectiveness of treatment. Participants can award a score of 7–9 for outcomes they find critical, 4–6 for outcomes they find important but not critical and 1–3 for outcomes of limited importance, as recommended by the Grading of Recommendations Assessment, Development and Evaluation (GRADE) working group [19, 23]. Scoring the outcomes by their relative importance results in a clearer picture of which outcomes are most important to the participants [24]. An ‘unable to score’ option will be made available in the survey to allow for participants who lack the expertise to score certain outcomes [17]. For each Delphi round, the participants will have 4 weeks to fill out the web-based survey and will receive a reminder via e-mail after 2 and again after 3 weeks if they have not yet filled out the survey. The e-mail in the third week will ask participants whether they are having difficulty completing the survey or whether they have decided not to participate in the study. The percentages of participants who completed each round will be documented separately for each stakeholder group. Only participants who have completed the survey are able to participate in the subsequent rounds.

Whether outcomes will be in- or excluded in the final COS will depend on the consensus. The outcomes will be classified as ‘consensus in’, ‘consensus out’ or ‘consensus to be further discussed’. Outcomes will be seen as essential and be classified as ‘consensus in’ if 70% or more participants have awarded a score of 7–9 to an outcome and less than 15% have awarded a score of 1–3 to that outcome [19]. If an outcome has been scored 7 or greater on average by the patients, the outcome will be classified as ‘consensus in’, regardless of the overall score of all participants, to ensure outcomes most important to the patients are included. Outcomes will be seen as unimportant and will be classified as ‘consensus out’ if 70% or more participants have awarded a score of 1–3 to an outcome and less than 15% have awarded a score of 7–9 to that outcome [19]. Any other combination of scores will be considered ‘consensus to be further discussed’ (Table 1) [19].

**Table 1:** Definition of consensus

Classification consensus	Criteria	Interpretation
Consensus in	≥ 70% of the participants rated the outcome 7–9 and less than 15% rated the outcome 1–3 OR the average patient rating is ≥ 7, regardless of other scores	Outcome is important
Consensus out	≥ 70% of the participants rated the outcome 1–3 and less than 15% rated the outcome 7–9	Outcome is not important
Consensus to be further discussed	All other results	Potentially important outcome

The results from the Delphi rounds will be shared to the group in each subsequent round, enabling participants to consider different opinions and review their original answers, which is expected to result in convergence towards a consensual opinion [25]. Consensus is not expected to be reached until the third round; however, if consensus has been reached after round two, a third round will not be necessary.

##### Delphi round 1

In the first round, participants will be sent an e-mail with background information and the rationale behind the development of the COS, along with a link to the web-based survey (Qualtrics). The survey provider will store names and e-mail addresses of the participants which will be used to later identify the participants who completed the Delphi rounds. In the first round, participants will be asked to state up to three outcomes they believe are the most important outcomes when evaluating treatment effectiveness. Participants will then be asked to rate the importance of the outcomes which were established through the systematic review and patient interviews on a 9-point Likert scale. In the final step of the survey, participants will be asked to list any outcomes they feel are important but were not listed and will be asked to submit any additional feedback on the survey.

Any new outcomes proposed by the participants in round 1 will be evaluated by the COS steering group to ensure they truly represent new outcomes. These new outcomes will then be put forward in the next round along with any ‘consensus to be further discussed’ outcomes.

At the end of the first Delphi round, the COS steering group will give a consensus classification to each outcome using the predefined classification criteria (Table 1).

##### Delphi round 2

The results from round 1 will be summarised and made anonymous. Any ‘consensus to be further discussed’ outcomes along with any newly identified outcomes put forward by participants during round 1 will be sent to all participants who completed round 1. Participants will also be sent the answers they provided in round 1 to compare their own answers to the answers of the group. This will allow the participants to adjust their answers if they wish to do so, which will likely lead to a convergence towards a consensual opinion [25]. At the end of the second Delphi round, the COS steering group will give a consensus classification to each outcome using the predefined classification criteria (Table 1).

##### Delphi round 3 (optional)

A third Delphi round may take place if consensus is not yet reached after round 2 or if many additional outcomes were presented by participants in round 1.

### Step 3: Consensus meeting

Following the Delphi rounds, a consensus meeting will be conducted with the COS steering group and representatives from each stakeholder group. During this meeting, the results of the survey will be discussed to agree on a final core outcome set. The outcomes classified as ‘consensus to be further discussed’ will be discussed as a group to classify the outcomes as either ‘consensus in’ or ‘consensus out’. To keep anonymity, the participants will vote through an anonymous online programme whether they believe the outcome should be included in the COS or not. The results will then be shown to all participants. Any further disagreement regarding an outcome which has been classified as ‘consensus in’ or ‘consensus out’ can also be discussed. A final Core Outcome Set will be agreed upon by the COS steering group at this consensus meeting.

## Discussion

At present, there is no Core Outcome Set for FI. The aim of this project is to develop a COS for FI so that there will be a standardised minimum set of outcomes which researchers should report on in FI intervention studies. A consistent set of outcomes in all FI intervention studies will enhance comparison between studies which will simplify the assessment of the effectiveness of therapeutic modalities. This in turn will improve the use of evidence-based treatment by medical professionals. Furthermore, the inclusion of patients in the development of the COS ensures that treatment algorithms better align with outcomes important to patients, thereby improving patient care. After creation of the COS, future research should focus on how the outcomes included in the COS should be measured. This can be determined through evaluation of different potential outcome measurement instruments. The selection of outcome measurement instruments to determine how an outcome should be measured is a multi-step consensus-based process similar to the selection of a COS, as outlined by the Consensus-based Standards for the selection of health Measurement Instruments (COSMIN) initiative [26]. After a list of outcome measurement instruments has been determined, distribution of the COS should take place. We envision that distribution in the future will mainly be conducted digitally through the communication channels of relevant scientific societies and also through presentations at relevant conferences.

## Data Availability

Not applicable
